# Practical guidelines for producing non-replicating canine adenovirus vectors

**DOI:** 10.1371/journal.pone.0341642

**Published:** 2026-05-20

**Authors:** Denis Omara, Christian Ndekezi, Susan Mugaba, Angella Nakyanzi, Henry Bukenya, Josephine Bwogi, Fortunate Natwijuka, Anne Kapaata, Frank Kato, Drake Byamukama, Mercy Lorna Ayebale, David Patrick Kateete, Jennifer Serwanga, Stephen Cose, Sande James Obondo, Pontiano Kaleebu, Sheila Nina Balinda

**Affiliations:** 1 Uganda Virus Research Institute (UVRI), Entebbe, Uganda; 2 Department of Immunology and Molecular Biology, School of Biomedical Sciences, College of Health Sciences, Makerere University, Kampala, Uganda; 3 Medical Research Council/Uganda Virus Research Institute & London School of Hygiene and Tropical Medicine (MRC/UVRI & LSHTM) Uganda Research Unit, Entebbe, Uganda; Janssen Vaccines & Prevention B.V., NETHERLANDS, KINGDOM OF THE

## Abstract

Adenovirus vectors have been used for vaccine development, gene therapy, among others, because of their ease of genetic manipulation and high efficiency in delivering and expressing genes in mammalian cells without causing significant cytotoxicity or integrating into the host genome. Adenovirus vectors are relatively immunogenic, hence avoiding the need for adjuvant when used in vaccine design, unlike other vaccine development platforms. Human adenovirus serotype 5 (HAdV-5) has traditionally been employed in vaccine platforms; however, its clinical utility is limited by widespread pre-existing immunity in human populations, which can reduce vaccine efficacy. As a promising alternative, canine adenovirus type 2 (CAV-2) vector offers several advantages, including low seroprevalence in humans, efficient infection of respiratory epithelial and neuronal cells, and long-lasting transgene expression. These features render the CAV-2 vector particularly suitable for vaccine applications requiring repeated administrations or booster doses. In this protocol, we describe methods for the expression, expansion, purification, and titration of a non-replicative CAV-2 vector in the AD-293 cell line. The procedure involves the release of the recombinant viral genome from the pUC19 plasmid backbone, followed by virus stock expansion in a complementing AD-293 cell line, which is able to express the E1 protein to aid virus replication. The virus stock was purified through ion-exchange liquid chromatography and titered using the Improved Kärber method. This work contributes to the growing field of alternative adenoviral vectors with implications for translational research by providing step-by-step procedures for producing a canine adenovirus type 2 (CAV-2) vector.

## 1. Introduction

Adenoviruses are medium-sized, non-enveloped, DNA viruses with linear double-stranded DNA genomes of approximately 36kb [1]. The virion has an icosahedral capsid structure measuring 70–90 nm in diameter, consisting exclusively of protein and DNA. Adenoviruses have been isolated from a range of mammalian and fowl species, and over 100 serotypes have been described to date, including more than 50 that infect humans, termed human adenovirus (HAdVs) [2].

Human adenoviruses (HAdVs) and canine adenoviruses (CAVs) belong to the Adenoviridae family [2] but differ significantly in their host specificity, pre-existing immunity profiles, and utility as viral vectors [3,4]. HAdVs, particularly serotypes like HAdV-5, have been widely used in gene therapy and vaccine platforms; however, their effectiveness can be hampered by high levels of pre-existing neutralising antibodies in the human population [5]. In contrast, canine adenovirus type 2 (CAV-2) is a non-human adenovirus with minimal seroprevalence in humans, making it an attractive alternative for vaccine delivery [2]. CAV-2 has a natural tropism for respiratory epithelial cells and neurons, and demonstrates efficient transgene expression and long-term immunogenicity [6]. Its low cross-reactivity with human adenoviral immunity reduces the likelihood of vector neutralisation upon administration [7]. These features make CAV-2 a promising platform for the development of vaccines, especially in scenarios where repeated dosing or booster vaccinations may be required.

Adenovirus vectors have gained tremendous attention as gene-delivery vehicles, vaccine vectors and oncolytic viruses [8,9]. Adenovirus vectors have numerous advantages: they can infect a wide variety of dividing or nondividing cells; they are easily purified to high titres; the strains commonly used to construct recombinant viruses are well characterized; they can accommodate up to 37 kb of foreign genetic material; their genomes can easily be manipulated; their genome rarely integrates into the host chromosome; and they are genetically stable, making them suitable for transient gene expression applications [9,10]. Importantly, Adenovirus vectors infect dendritic cells, upregulate co-stimulatory molecules, and elicit cytokine and chemokine responses, thus effectively presenting antigens to the immune system and eliciting potent immune responses [11,12].

Adenovirus vectors can be designed either as replication-competent or replication-defective, and a broad spectrum of replicating and non-replicating adenovirus vectors has been developed [9]. The choice of the vector depends on the biology of the infectious agent targeted, as well as factors such as whether the vaccine is intended to prevent infection or boost immunity in already infected individuals, prior exposure of the target population to the vector, safety, and the number and size of gene inserts needed [9]. Adenovirus vectors are rendered replication-defective by deletion of the E1 region genes, essential for replication [9]. Such vectors generally have the non-essential E3 region deleted as well, in order to create more space for foreign genes [9]. An expression cassette is then inserted with the transgene under the control of an exogenous promoter [9]. The CAV-2 vector used in this work was obtained from the Institut de Génétique Moléculaire de Montpellier (IGMM). The vector was engineered by deleting the E1 and E3 regions from the virus genome to prevent replication and increase cloning capacity. The vector carries a cytomegalovirus (CMV) promoter that leads to Dre recombinase expression. Several publications have described the generation of recombinant CAV-2 plasmids, and these remain foundational for current vector-design strategies. Early methodological frameworks (e.g., using homologous recombination in E. coli between a plasmid bearing the full CAV-2 genome and a shuttle plasmid carrying a transgene cassette) enabled deletion of the E1 region and insertion of heterologous expression cassettes, followed by virus rescue in complementing cell lines [2]. In recent years, these approaches have been refined and optimized: for example, a comprehensive review in 2019 provides updated protocols for CAV-2 vector construction and emphasizes the use of improved bacterial recombination systems for seamless insertion of expression cassettes [6]. Together, these works define best practices for designing and constructing recombinant CAV-2 genomes suitable for preclinical use.

After generating the recombinant adenovirus plasmid by cloning, the viral genome must be released from the plasmid for virus expression and expansion in an appropriate cell line. In this protocol, we have described methods for expressing and expanding the non-replicative canine adenovirus vector type 2 (CAV-2) in the AD-293 cell line. We also describe the purification and titration procedures for the vector.

## 2. Methods and results

**Fig 1:**
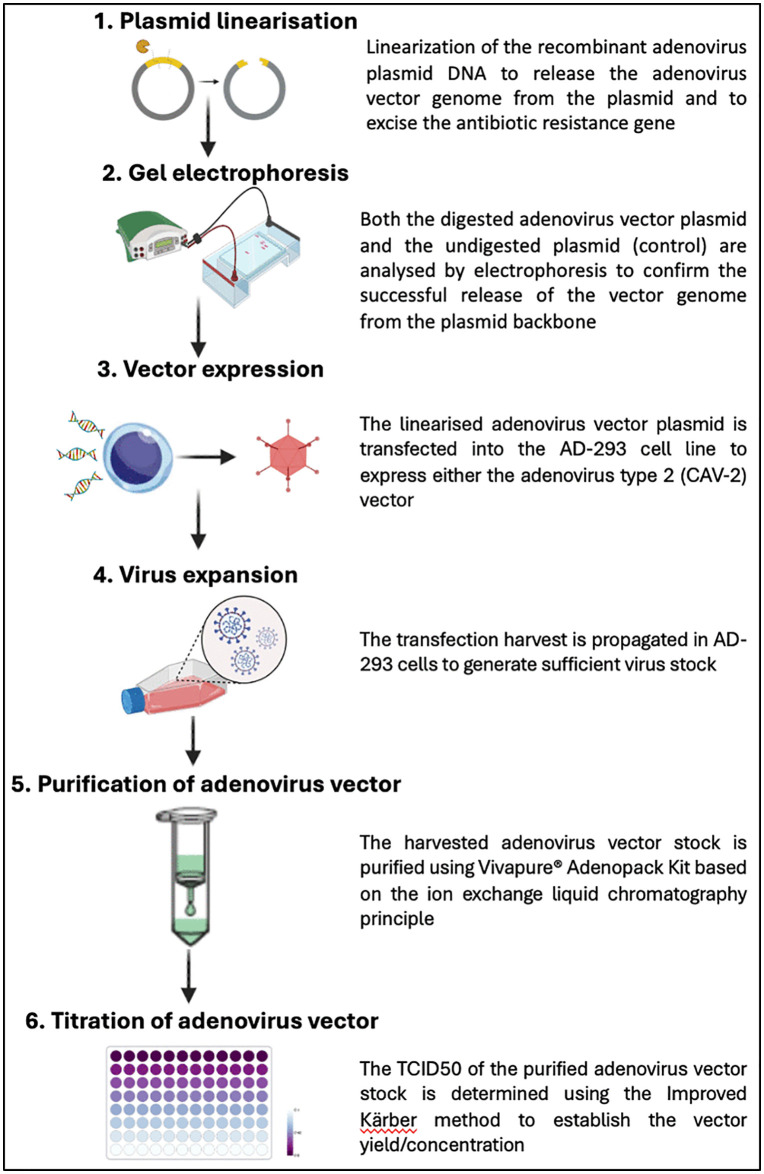
A schematic overview of the laboratory workflow in the generation of the non-replicative adenovirus vector. The figure was generated using components from bioRender.

Briefly, the laboratory procedures involve the release of the recombinant CAV-2 vector genome from the pUC19 plasmid backbone by conducting restriction digestion and transfecting it into the AD-293 cell line for expression, followed by virus stock expansion also in the AD-293 cell line. The AD-293 cell line is a complementing cell line which is able to express the E1 protein to facilitate virus replication since the vector is E1-deleted. The virus stock was purified by ion-exchange liquid chromatography using the Vivapure® Adenopack™ 20 Kit and titered using the Improved Kärber method by determining the 50% tissue culture infectious dose (TCID50), which provides a more direct count of infectious virus particles as illustrated in **Fig 1**. The information on the specifications and the sources of the reagents, consumables and equipment, as well as recipes of the solutions used in this protocol, can be found in S2-5 Tables, respectively. The protocol described in this peer-reviewed article is published on protocols.io, https://dx.doi.org/10.17504/protocols.io.5qpvo131dg4o/v1 and is included for printing as supporting information file 1–3 with this article. This study was conducted according to the guidelines of the Declaration of Helsinki. Although this work itself did not require an ethics approval since the materials (CAV-2 vector and AD-293 cell line) used were from commercial sources, the study was part of a bigger study that was approved by the Uganda Virus Research Institute Research and Ethics Committee (UVRI-REC) (Ref: GC/127/20/12/803), School of Veterinary Medicine and Animal Resources Institutional Animal Care and Use Committee (Ref: SVAR-IACUC/60/2020) and registered with the Uganda National Council for Science and Technology (UNCST)(Ref: HS1153ES).

### 2.1. Linearization of the recombinant adenovirus plasmid DNA to be used in transfection

Adenovirus packaging systems like HEK293-based systems typically require linear DNA for efficient rescue and packaging of the recombinant virus [13]. Most recombinant adenovirus plasmids are maintained in circular form in bacteria for stability, but they need to be cut at a specific site, often PacI or another unique site, to mimic the natural viral genome structure before being introduced into mammalian cells.

The recombinant adenovirus plasmid usually contains a bacterial backbone used for propagation in *E. coli,* including antibiotic resistance genes (e.g., ampicillin or kanamycin resistance) and bacterial origin of replication. These elements are non-essential and potentially harmful if carried into mammalian cells. Restriction digestion removes these sequences, ensuring: better biosafety (i.e., antibiotic resistance genes are not introduced into human/animal cells) [14], improved transgene expression since bacterial elements can suppress transcription, and higher purity of viral genome for packaging.

Linearised adenoviral DNA allows the cellular machinery to properly recognize the Inverted Terminal Repeats (ITRs), which are crucial for initiating replication and packaging the genome into capsids [13]. If the plasmid is not linearized, packaging might fail or be highly inefficient [13]. Circular plasmids may lead to non-homologous recombination and rearranged or defective viral genomes [15]. This could result in non-functional or replication-deficient viruses that do not express your gene of interest properly. Therefore, Restriction digestion serves as a quality check to confirm that the plasmid has the correct insert and that no major rearrangements or contaminations have occurred. Maintaining aseptic conditions is essential throughout the process of linearising circular plasmid DNA.

Use the *PacI* restriction enzyme (NEB, Cat. R0547S) to cleave the recombinant adenovirus plasmid to release the intact adenovirus vector genome. The PacI sites flank both 5′ and 3′ ITRs located at the ends of the adenovirus vector genome. In a 1.5 mL Eppendorf tube placed on ice, set up the digest reaction using 1 µg of recombinant adenovirus plasmid DNA, 0.2 μL of PacI restriction enzyme, 1 μL of CutSmart^TM^ buffer (10X) and top up the reaction volume to 10 μL using nuclease-free water. Incubate the digest reaction for 60 min at 37°C and terminate the reaction by denaturing the enzymes at 65°C for 10 minutes.

To confirm the successful release of the CAV-2 vector genome from the plasmid backbone, analyse 1 μL of digested plasmid using electrophoresis on a 1% agarose gel.

### 2.2. Transfection of AD-293 cells with linearised adenovirus vector DNA to express the virus vector

Transfection is the process of deliberately introducing foreign nucleic acids, in this case, the linearised adenovirus vector DNA, into eukaryotic cells [16]. This enables the expression of the recombinant adenovirus in viral particle form. In this protocol, the AD-293 cell line (Cat 240085, Agilent, U.S.A.) was used. AD-293 cells are a derivative of HEK293 cells, optimised for high transfection efficiency and adenovirus production. These cells express adenoviral E1 genes, which are essential for rescuing and propagating E1-deleted adenovirus vectors. Once inside the cells, the linearised viral genome is recognised by the host cell machinery. The virus is replicated, assembled, and released into the culture medium as recombinant adenovirus particles. The step-by-step transfection procedure can be conducted as described below.

One day before transfection, seed AD-293 cells in a 6-well culture plate at 0.5 x10^6^ cells per well in 2 mL of 2% cDMEM and incubate the plate at 37°c and 5% CO_2._

When cell confluency reaches 50% to 70% on the next day, proceed with transfection.

For each transfection, label two 0.5-ml Eppendorf tubes as tubes A and B. Add 1 µg of PacI-digested recombinant adenovirus plasmid DNA to tube A and adjust the total volume to 60 µL with plain DMEM. Add 6.4 µL of Lipofectamine and 53.6 µL of plain DMEM to tube B. Gently mix the contents of each tube separately by swirling the tubes. Do not vortex. Combine the solutions of tubes A and B using a wide-bore pipette and mix them gently by swirling. Allow the mixture to incubate at room temperature for 45 minutes to enable the formation of DNA–liposome complexes.

Five minutes before the incubation ends, gently wash the cells once with 2 ml of plain DMEM pre-warmed to 37°C, then add 3 ml of DMEM without serum and antibiotics. Add the transfection mixture to the cells dropwise, rock the plate gently, and return the cells to the incubator. Three hours later, add 60 µL of FBS and incubate the cultures overnight.

**Fig 2:**
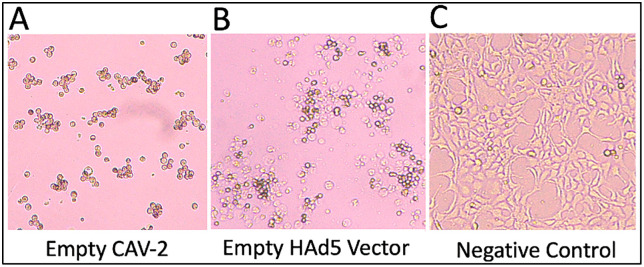
Cytopathic effect formation post-transfection of AD-293 cells with CAV-2 vector (A), human adenovirus vector as a positive control (B) and a mock test as a negative control (C). (Olympus CKX53) at all the three images taken at 100× and 200 × magnifications.

The next day, remove the transfection medium and replace it with fresh complete growth medium (2% cDMEM), then continue incubating the transfected cells. Add 200 μL of fresh Complete growth medium to each well every 3 days and examine the cells for cytopathic effect (CPE). CPE reflects the morphological changes of the cultured cells as infection proceeds. Such distinctive morphological changes result from the accumulation of newly produced virus proteins and progeny virions which disrupt normal cell structure resulting into cell death. CPE is different from necrosis; CPE begins with the rounding of adhered cells, followed by the gradual detachment of the rounded cells from the plate. When the cells detach from the plate, they may form “grape-like” clusters that float in the growth medium (Fig 2: A and B).

When 90% of the cell monolayer has shown cytopathic effects (CPE), detach any remaining cells by either gently tapping the plate on the biosafety cabinet surface or by pipetting the medium against the growth surface of the well. Transfer the cell suspension to a 2-ml cryogenic vial and store at −80°C for virus expansion. If the transfected cells do not show any sign of CPE 1 week after the transfection, see the discussion 3.1.

### 2.3. Expansion of adenovirus vector for large-scale use

After successfully rescuing a recombinant adenovirus vector usually by transfecting linearised vector DNA into packaging cells like AD-293, the next step is virus expansion to produce larger amounts of the virus for experimental use, preclinical studies, or production scale-up. In the case of vaccine development and formulation, sufficient virus titers can be achieved through virus vector expansion, ensuring batch consistency and reproducibility of vaccine evaluation experiments. The adenovirus vector expansion can be conducted as described below.

Seed 10 X 10^6^ AD-293 cells into a T75 cell culture flask in a total volume of 19 ml of 2% cDMEM, a day before viral infection.

Thaw the cell suspension obtained in step 2.2.6 in a 37°C water bath to generate a crude cell lysate. Repeat the freeze–thaw cycle twice more in a dry-ice/ethanol bath and a 37°C water bath. Shake the tube several times after each thaw to ensure that cells do not settle.

Following three freeze-thaw cycles, spin the crude lysate at 3200 rpm for 10 minutes at 4°C, then retrieve the supernatant to infect AD-293 cells cultured in a T75 flask in step 2.3.1.

Add 1 mL of the supernatant directly to 70%–80% confluent T-75 flask of AD-293 cells. Return the cells to the incubator and monitor CPE daily (Fig 2). Usually, CPE will become noticeable 24 hours after infection and will be fully evident within 2–3 days.

Once approximately 90% of the cells show cytopathic effects (CPE), collect them as previously described. With the flask lid secured, gently agitate the culture and use a 10-ml pipette to dislodge any remaining adherent cells. Transfer the harvest into sterile 50-ml conical centrifuge tubes.

Spin the cells at 3200 rpm for 10 minutes at 4°C to form a pellet. Carefully remove the supernatant and resuspend the pellet in 2 mL of 2% complete DMEM.

Freeze-thaw the cell pellet as described in step 2.3.2 to release the cell-bound virus. After three cycles of freezing-thawing, centrifuge the crude cell lysate at 3200 rpm for 10 minutes at 4°C and collect the supernatant. Add this supernatant to the previously collected one and store at −80°C or proceed with the purification method. If the crude viral lysate harvested from the transfection step fails to expand after 1 week, see discussion 3.2.

### 2.4. Purification of the Adenovirus Vectors using Vivapure® Adenopack™ 20 Kit

Purification of adenovirus vectors is a critical step after virus stock production. It removes host cell proteins, DNA, and debris to yield a high purity. In this protocol, the virus stock was purified using the Vivapure® Adenopack™ 20 kit based on the ion-exchange liquid chromatography principle. This technique offers high specificity and scalability for separating viral particles based on surface charge. Compared to conventional methods such as ultracentrifugation or sucrose/Caesium Chloride (CsCl) density gradient purification, it offers a rapid, reproducible, and scalable method suitable for small-to-medium preclinical vector preparations, and it avoids the extended ultracentrifugation steps required for CsCl gradients [17–20]. Additionally, it reduces the risk of viral particle degradation due to lower shear forces and avoids the use of toxic gradient materials like Caesium Chloride (CsCl) [19]. The Vivapure Q Maxi M spin columns in the Vivapure® Adenopack™ 20 kit has an ion exchange membrane adsorber that binds adenoviral particles. Once bound, virus particles can be further purified by washing away non-specifically bound proteins before elution within one hour. Each Vivapure Q Maxi M spin column is suitable for purifying virus from culture volumes of up to 20 mL. Reagent volumes can be adjusted accordingly for smaller samples. This kit contains sufficient consumables for six such small preparations.

#### 2.4.1. Sample preparation.

Add 1µl Benzonase® for each 1 ml culture volume, to a final concentration of 12.5U/ml. E.g., if 20 ml (T75 culture flasks) were used to cultivate the virus, add 20µl Benzonase®.

Thoroughly mix the sample and incubate at 37°C for 30 minutes to enzymatically degrade cellular nucleic acids.

Transfer the digested supernatant onto a Vivaclear Maxi filter unit and centrifuge at 500 × g for 5 minutes or until the entire volume has filtered through the membrane.

Retrieve the flow-through, approximate its volume, and gradually add one-ninth of that volume of the 10 × loading buffer while gently agitating to prevent osmotic shock to the viral particles. E.g., 2 ml to 18 ml flow-through. Accurate volume measurements of the supernatant and 10X Loading Buffer are critical to achieving the right conditions for binding virus particles.

#### 2.4.2. Vivapure Q maxi M preparation.

Prepare the working solution by diluting the 10 × Washing Buffer, for example, by mixing 5 ml of buffer with 45 ml of deionised water, and ensure thorough mixing.

To equilibrate the Vivapure Q Maxi M spin column, add 5 ml of diluted Washing Buffer and centrifuge at 500 × g for 5 minutes. To promote consistent sample flow, using a swing-out rotor is advisable.

#### 2.4.3. Adenovirus purification.

Add up to 20 ml of the sample to the Vivapure Q Maxi M insert and centrifuge at 500 × g for 5 minutes or until the entire volume has passed through the membrane. Collect the flow-through and, if needed, repeat the process with the remaining sample.

Rinse the spin column by centrifuging with 18 ml of Washing Buffer at 500 × g for 5 minutes. Discard the flow-through and repeat the wash step a second time.

Transfer the spin column to a new collection tube and elute the adenovirus from the Vivapure Q Maxi M membrane with 1 ml of Elution Buffer. Pipette buffer onto the membrane, centrifuge for 30 s at 500 x g and incubate for 10 min. Then spin the device for 5 minutes at 500 x g and collect the adenovirus-containing eluate. Applying a second elution step could improve yield, but it will dilute the virus titre.

#### 2.4.4. Optional: Buffer exchange and concentration.

It is necessary that the virus is exchanged into the physiological buffer before use in tissue culture or cell-based assays, or into the generic storage buffer for long-term storage at −80°C [21]. Storage Buffers containing glycerol may take considerably longer to concentrate than the original viral eluate solution; prolong centrifuge times, and, if necessary, use cooling at +4°C.

Pour the eluate into a Vivaspin 20 centrifugal concentrator and add storage or physiological buffer to adjust the total volume to 10 ml.

Centrifuge for 30 minutes at 800 x g in a swing-out rotor, or 25° fixed-angle rotor, with cavities accepting 50 ml conical bottom tubes.

Check the volume of viral concentrate remaining in the upper chamber and, if necessary, centrifuge again and repeat buffer exchange a second time. Do not reduce the volume to less than 200 µl to avoid aggregation and loss of infectivity.

Recover the concentrated virus using by pipette. Resuspend concentrated virus by gently pipetting up and down a few times before recovery.

Determine viral titre. Aliquot accordingly and store the virus at –80°C.

### 2.5. Titration of the purified virus stocks using the Improved Kärber method

Titration of purified virus stocks is a crucial step in quantifying the infectious units within a viral preparation. In this protocol, the purified virus stock was titered using the Improved Kärber method, a robust and widely accepted statistical approach for determining the median (50%) tissue culture infectious dose (TCID₅₀) [22]. Unlike plaque assays, which require virus-induced cytopathic effects (CPE) to form discrete plaques, the Improved Kärber method accommodates viruses that produce diffuse or subtle CPE. It is also less time-consuming and easier to interpret in cases where plaque morphology is inconsistent. Furthermore, the Improved Kärber method requires fewer replicates and less precise quantification of individual plaques, making it a practical choice for rapid and reliable viral titration in early-phase studies. This method ensures accurate titration, which is essential for standardising experiments and ensuring reproducibility in virological studies. In this study, we titered the purified CAV-2 vector virus stock.

The Improved Kärber method involves preparing a monolayer of AD-293 cells in 96-well plates, followed by a series of dilutions of the virus stock. The endpoint, where 50% of wells show CPE, helps calculate the tissue culture infectious dose (TCID50), providing a reliable measure of viral infectivity.

#### 2.5.1. Cell preparation, virus serial dilution and CPE observation.

A day before, trypsinise and count AD-293 cells. Then prepare 20 ml of 1000 cells/ml in 10% cDMEM culture medium.

Dispense 100μl (~100 cells) per well in two 96-well plates per sample and put them in an incubator.

On the following day, prepare virus dilutions by adding 108μl of 10% cDMEM to all the wells of 96 U-bottom dilution plates from column 1 to column 10 for ten replicas. Columns 11 and 12 are control columns, where 100uL of culture medium should be added.

Retrieve purified virus stocks from −80 ^0^C freezer, thaw and add 12uL of the virus stock to the first row to make 10^−1^ (10-fold) dilution.

Using a multichannel pipette, serially dilute the virus stock by transferring 12uL from row 1 (10^-1^) to row 2 (10^-2^) and continue up to row 16 (10^-16^) on the second plate.

Retrieve the culture plates that have already attained about 70% confluency from the incubator and remove the medium from the wells.

Transfer 100 µL of the diluted virus from the dilution plate to the culture plate in their respective wells and incubate at 37°C with 5% CO_2_.

Score wells for cytopathic effects (CPE) by counting the wells that have shown CPE (Fig 2) daily until there is no more CPE formation for two consecutive days. For the experiment to be valid, all the control wells must show no CPE.

### 2.5.2. Calculation of 50% Tissue Culture Infectious Dose (TCID50) of the titered virus stocks

Calculate the titres by determining the ratio of positive wells per row, as shown in Table 1, and use the Improved Kärber formula to determine the TICD50 [22].

**Table 1:** Titration of the purified canine adenovirus type 2 (CAV-2) vector virus stock.

10-fold Dilution	1	2	3	4	5	6	7	8	9	10	Control		Total CPE Ratio	Key	
10^−1^	0.1	0.1	0.1	0.1	0.1	0.1	0.1	0.1	0.1	0.1	NC	NC	1	**Days Post** **Infection**	**No. of Wells Showing CPE**
10^−2^	0.1	0.1	0.1	0.1	0.1	0.1	0.1	0.1	0.1	0.1	NC	NC	1		
10^−3^	0.1	0.1	0.1	0.1	0.1	0.1	0.1	0.1	0.1	0.1	NC	NC	1	CPE on Day 1	10
10^−4^	0.1	0.1	0.1	0.1	0.1	0.1	0.1	0.1	0.1	0.1	NC	NC	1	CPE on Day 2	8
10^−5^	0.1	0.1	0.1	0.1	0.1	0.1	0.1	0.1	0.1	0.1	NC	NC	1	CPE on Day 3	38
10^−6^	0.1	0.1	0.1	0.1	0.1	0.1	0.1	0.1	0.1	0.1	NC	NC	1	CPE on Day 4	20
10^−7^	0.1	0.1	0.1	0.1	0.1	0.1	0.1	0.1	0.1	0.1	NC	NC	1	CPE on Day 5	10
10^−8^	0.1	0.1	0.1	0.1			0.1	0.1	0.1	0.1	NC	NC	0.8	CPE on Day 6	0
10^−9^	0.1	0.1		0.1			0.1	0.1		0.1	NC	NC	0.6		
10^−10^	0.1			0.1				0.1		0.1	NC	NC	0.4	LogTCID_50_ =	−15.3
10^-11^											NC	NC	0	TCID_50_/100uL =	1/10^-15.3^
10^-12^											NC	NC	0	The titer, given a viral inoculum of 0.1 mL, is therefore:	
10^-13^											NC	NC	0	TCID_50_/mL =	(1/10^-15.3^)/0.1
10^-14^											NC	NC	0	TCID_50_/mL =	2.0 x 10^16^
10^-15^											NC	NC	0		
10^-16^											NC	NC	0		
												**Total**	**8.8**		


$$\begin{array}{c} \text{Improved K}\ddot{\text{a}}\text{rber Formular}=\text{log}{\text{TCID}}_{\text{50}}=\text{log}d_{L}-\text{log}d_{F}\left(\sum p_{i}-\text{0}.\text{5}\right) \end{array}$$

Where:

*d*_*L*_ = lowest dilution at which all wells are positive of which in this case is 10^−7^ (**Table 1**)

*d*_*F*_ *=* dilution factor of which in this case is 10-fold (**Table 1**)

$\sum p_{i}$*=* sum of the proportion of positive wells for all dilutions showing CPE of which in this case is 8.8 (**Table 1**)

## 3. Discussion

### 3.1. Transfection

In certain instances, AD-293 cells that have been transfected exhibit no observable cytopathic effects even after seven days post-transfection. This can be troubleshooted by repeating the transfection using an alternative transfection reagent like FuGene (Cat. E2311, Promega) and/or extending the incubation time up to 2 weeks post-transfection and/or increasing the amount of transfection DNA [23]. It is essential to include positive and negative controls in the transfection assay to aid accurate interpretation of the observations. A positive control, such as a vector known to express efficiently, confirms that the transfection reagents, cell line, and protocol are working as expected. If the positive control fails, it indicates a problem with the transfection conditions rather than the test vector construct. A negative control, on the other hand (e.g., a mock transfection or a plasmid lacking the gene of interest), helps establish a baseline for background expression or toxicity, ensuring that observed effects are due to the specific vector being tested and not due to non-specific effects or reagent toxicity. Together, these controls provide a reference framework, improve reproducibility, and enhance the reliability of the experimental conclusions.

### 3.2. Expansion of Virus stocks

The viral lysate may fail to expand in AD-293 cells. Several factors may contribute to this issue. These include cytotoxic effects resulting from transgene expression, which can lead to cell death rather than the development of viral cytopathic effects (CPE) [24]; an oversized transgene cassette [25]; and variations in the type and extent of early gene deletions within the vector backbone (such as deletion of E1 alone, E1/E3, or E1/E3/E4 regions) [26]. Additionally, the expression levels of complementing genes in the host cell line, such as E1 expression in AD-293 cells, used to support replication of replication-defective adenoviral vectors, can influence outcomes [27]. Another contributing factor is asynchronous infection due to a low multiplicity of infection (MOI) [28]. In such cases, especially when cells are infected at low MOIs, serial passaging of the virus may be necessary to achieve complete CPE [26]. Moreover, for vectors that exhibit a slow and delayed infection process, it becomes challenging to distinguish between virus-induced CPE and cell death caused by other factors. To address this issue, several strategies can be employed. First, designing an inducible transgene expression cassette to regulate the expression of potentially toxic transgenes in AD-293 cells, thereby minimising cytotoxicity [24]. Additionally, the expansion process should be slowed down; this involves initiating the expansion from the crude lysate obtained during the initial transfection or rescue phase by infecting AD-293 cells in a T25 flask, and subsequently scaling up to a T75 flask [26]. If, during this expansion phase, full cytopathic effect (CPE) is not observed within one week, the entire infected culture should be harvested, the crude lysate clarified, and used to initiate the next round of infection [26].

### 3.3. Purification of adenovirus vector

It’s advisable to first filter the digested supernatant through a 0.22μm filter to remove most of the host cell debris before loading the digested supernatant on a Vivaclear Maxi to avoid clogging the Vivaclear Maxi membrane. Besides the Vivapure® Adenopack™ 20 Kit that purifies 20 mL culture volume, there are other kits, for example, Vivapure Adenopack™ 100, Cat. VS-AVPQ101 and Vivapure Adenopack™ 500, Cat VS-AVPQ501, which can be used to purify bigger culture volumes: 200 ml culture volume and 500 ml culture volume, respectively. In addition to the ion exchange liquid chromatography method of purifying virus stocks, there are also other methods of liquid chromatography, which include normal phase, reverse phase and size exclusion that can be explored [29]. Besides the liquid chromatography principle, density gradient centrifugation is another commonly used principle for purifying virus stocks: these include rate zonal centrifugation and isopycnic equilibrium gradient centrifugation [30]. While several reports indicate that membrane-based ion-exchange purification can yield high-quality adenoviral preparations with reduced processing time [17,31,32], we acknowledge that direct, side-by-side purity comparisons between Adenopack-purified CAV-2 and CsCl-purified CAV-2 are limited.

We used the Vivaspin 20 units because our workflow required a rapid buffer exchange into the physiological storage buffer and a small final volume compatible with downstream analyses (i.e., the achieving the high dose in small volume). While alternative approaches (e.g., diafiltration, desalting columns) can also perform buffer exchange, they either require larger starting volumes, introduce longer processing times, or provide less precise control over final concentration [33]. For these reasons, the Vivaspin concentrators were the most practical choice for our small-scale preclinical preparations.

### 3.4. Titration

During the titration process, in some cases, no CPE may be observed in all the wells two weeks post-inoculation, even in the wells containing the highest concentration of virus stock (10^-1^ dilution). This could be due to low virus stock titre or the virus stock being non-infectious. Therefore, it is advisable that the titration experiment be repeated using a lower dilution factor instead of a 10-fold dilution factor or troubleshooting the virus stock by running a positive control virus of known infectious stock and a mock control on the same plate [34]. This immediately tells you whether the assay/cells are working. Check cell confluency, passage number, viability and health by testing for mycoplasma; unhealthy/contaminated cells often fail to show CPE [35–37]. On the other hand, all wells may show CPE, causing uncertainty on the dilution endpoint. Therefore, it is advisable that the titration experiment be repeated with a high dilution factor instead of a 10-fold dilution factor [38].

### 3.5. Biosafety guidelines

The NIH Guidelines for Research Involving Recombinant DNA Molecules classify both wild-type and replication-deficient human adenoviruses as biohazard risk group 2 agents [39]. The human diseases caused by these biohazard agents are preventable, treatable, and generally mild [40]. Handling of adenoviral vectors should occur within a Biosafety Level 2 (BL2) facility and must be approved by the Institutional Biosafety Committee (IBC) of the respective institution.

Personnel should use appropriate personal protective equipment (PPE), including lab coats, gloves, and eye protection. Work should be performed inside a certified biological safety cabinet (Class II BSC) to minimise the risk of aerosol exposure. Special attention should be paid to preventing spills and accidental release of the virus, and any spills must be immediately disinfected using an approved disinfectant effective against adenoviruses, such as a freshly prepared 10% bleach solution.

During purification procedures, particularly when using high-speed centrifugation (e.g., density gradient ultracentrifugation), additional precautions should be taken to avoid the formation of infectious aerosols. Centrifuge rotors and buckets should be loaded and unloaded within a BSC, and rotors should be sealed during centrifugation. After centrifugation, equipment and surfaces must be carefully disinfected.

In the titration and handling of concentrated viral stocks, it is critical to minimise potential exposure risks. All viral stocks should be clearly labelled, securely stored within secondary containment, and waste must be decontaminated before disposal, typically by chemical disinfection or autoclaving. Accidental exposure incidents should be immediately reported and medically assessed according to institutional policies. Additionally, training in adenovirus handling, awareness of the symptoms of accidental exposure (e.g., conjunctivitis, respiratory symptoms, etc), and having an established medical surveillance plan for laboratory workers handling adenoviral vectors is highly recommended.

## Supporting information

S1 Table Reagents. (DOCX)

S2 Table Consumables. (DOCX)

S3 Table Equipment. (DOCX)

S4 Table Recipes. (DOCX)

S1 File SOP Transfection, infection, expansion, and large-scale production of modified Adenovirus using AD293 cell line. (PDF)

S2 File SOP Vivapure Adenopack 20 RT Virus Purification. (PDF)

S3 File SOP virus titration using Improved Kärber Method. (PDF)
