# Supplementary material for: Practical guidelines for producing non-replicating canine adenovirus vectors
Source: PLoS One. 2026 May 20;21(5):e0341642. doi: 10.1371/journal.pone.0341642 (PMC13189411; doi:10.1371/journal.pone.0341642)
Supplement: S1 Table — (DOCX) [file pone.0341642.s001.docx]

| **Item** | **Specification** | **Supplier** |
| --- | --- | --- |
| Canine adenovirus vector (CAV-2) | CAV Dre | Institut de Génétique Moléculaire de Montpellier (IGMM), Montpellier, France |
| PacI endonuclease | Cat. R0547S | New England Biolabs, Massachusetts, USA |
| CutSmart^TM^ buffer (10X) | Cat. B7204 | New England Biolabs, Massachusetts, USA |
| Nuclease free water | Cat. AM9260G | Invitrogen™, California, USA |
| Agarose gel (1%) | Cat. 16500500 | Invitrogen™, **California, USA** |
| Large-range DNA ladder | Cat. 419-136 | GeneON, **Rhein**, Germany |
| AD-293 cells | Cat. 240085 | Agilent, Santa Clara, California, USA |
| 6-well culture plate | Cat. 140675 | ThermoFisher Scientific, New York, USA |
| 10% cDMEM and 2% cDMEM | (S4 Table) |  |
| DMEM, high glucose, pyruvate | Cat. 41966029 | Gibco^TM^, New York, USA |
| Fetal bovine serum (FBS) | Cat. 10082147 | Gibco^TM^, USA |
| Penicillin-Streptomycin (10,000 U/mL) | Cat. 15140122 | Gibco^TM^, Waltham, USA |
| GlutaMAX^TM^ (200 mM) | Cat. 35050061 | Gibco^TM^, New York, USA |
| PBS, pH 7.4 | Cat. 10010023 | Gibco^TM^, New York, USA |
| HEPES, 1M Buffer Solution | Cat. 15630080 | Gibco^TM^, Scotland, UK |
| Trypsin-EDTA (0.25%), phenol red | Cat. 25200056 | Gibco^TM^, Scotland, UK |
| Lipofectamine™ 2000 Transfection Reagent | Cat. 11668019 | ThermoFisher Scientific, Scotland, UK |
| Vivapure® Adenopack™ 20 kit | Cat.VS-AVPQ020 | Progen Biotechnik, Heidelberg, Germany |
| Physiological buffer | (S4 Table) |  |
| Tris hydrochloride | Cat. T2663 | Sigma-Aldrich, **Darmstadt, Germany** |
| Sodium chloride solution | Cat. S8776 | Signa-Aldrich, **Darmstadt, Germany**. |
| Glycerol | Cat. 49767 | Signa-Aldrich, Darmstadt, Germany |
