# Supplementary material for: Practical guidelines for producing non-replicating canine adenovirus vectors
Source: PLoS One. 2026 May 20;21(5):e0341642. doi: 10.1371/journal.pone.0341642 (PMC13189411; doi:10.1371/journal.pone.0341642)
Supplement: S2 Table — (DOCX) [file pone.0341642.s002.docx]

| **Item** | **Specification** | **Supplier** |
| --- | --- | --- |
| 0.5-mL Eppendorf Tube | Cat. 022600001 | Eppendorf, Hamburg, Germany |
| 2-mL cryogenic vial | Cat. CLS431416 | Corning Inc., New York, USA |
| T-75 cell culture flask | Cat. 10364131 | Nunc^TM^, Roskilde, Denmark |
| 5mL Serological Pipette | Cat. 75816-094 | Avantor, Pennsylvania, USA |
| 10mL Serological Pipette | Cat. 75816-100 | Avantor, Pennsylvania, USA |
| 25mL Serological Pipette | Cat. 75816-090 | Avantor, Pennsylvania, USA |
| 10µL BT Barrier Pipette Tips | Cat. 63300747 | Avantor, California, USA |
| 20µL BT Barrier Pipette Tips | Cat. 63300757 | Avantor, California, USA |
| 200µL BT Barrier Pipette Tips | Cat. 63300762 | Avantor, California, USA |
| 1000µL BT Barrier Pipette Tips | Cat. 63300750 | Avantor, California, USA |
| Hemacytometer | Cat. 3048-12 | Weber Scientific, New Jersey, USA |
| 50-mL sterile conical centrifuge tubes | Cat. 1525-0609 | Avantor, USA |
