# Supplementary material for: Practical guidelines for producing non-replicating canine adenovirus vectors
Source: PLoS One. 2026 May 20;21(5):e0341642. doi: 10.1371/journal.pone.0341642 (PMC13189411; doi:10.1371/journal.pone.0341642)
Supplement: S3 Table — (DOCX) [file pone.0341642.s003.docx]

| **Item** | **Specification** | **Supplier** |
| --- | --- | --- |
| Electrophoresis system | - | Any company |
| Gel imager | - | Any company |
| Incubator (37^0^C 5% CO_2_, humid) | - | Any company |
| Inverted Microscope | - | Any company |
| -80^0^C freezer | - | Any company |
| Water Bath Incubator (37^0^C) | - | Any company |
| Dry-ice/ethanol bath | - | - |
| Refrigerated tabletop centrifuge | - | Any company |
