# Supplementary material for: Practical guidelines for producing non-replicating canine adenovirus vectors
Source: PLoS One. 2026 May 20;21(5):e0341642. doi: 10.1371/journal.pone.0341642 (PMC13189411; doi:10.1371/journal.pone.0341642)
Supplement: S4 Table — (DOCX) [file pone.0341642.s004.docx]

| - - 1. **cDMEM** | | | |
| --- | --- | --- | --- |
| **Reagent** | **Quantity for 500 mL** | | **Final concentration** |
| DMEM | 435 mL or 475 mL | |  |
| Fetal bovine serum (FBS) | 50 mL or 10 mL | | 10% or 2% |
| Penicillin-Streptomycin (10,000 U/mL) | 5 mL | | 100 U/mL |
| GlutaMAX^TM^ (200 mM) | 5 mL | | 2 mM |
| HEPES, 1M Buffer Solution | 5 mL | |  |
| *Store at 4^0^C.* |  | |  |
|  |  | |  |
| - - 1. **Physiological buffer** | | | |
| **Reagent** | |  | **Final concentration** |
| Tris/HCl | |  | 20 mM |
| NaCl | |  | 25 mM |
| Glycerol (w/v) | |  | 2.5% Glycerol |
| *Store at 22^0^C.* | |  |  |
