# Supplementary material for: Practical guidelines for producing non-replicating canine adenovirus vectors
Source: PLoS One. 2026 May 20;21(5):e0341642. doi: 10.1371/journal.pone.0341642 (PMC13189411; doi:10.1371/journal.pone.0341642)
Supplement: S1 File — (PDF) [file pone.0341642.s005.pdf]

MRC/UVRI and LSHTM Uganda Research Unit

Medical Research Council

Uganda Virus Research Institute

LONDON SCHOOL of HYGIENE &TROPICAL MEDICINE

SOP Transfection, infection, expansion, and large-scale production of modified Adenovirus using AD293 cell line	Version 1.0	Effective Date 30-March-22

DO NOT COPY

Written by:	Name: Omara Denis<br>Function/role: Laboratory technologist

APPROVAL OF STANDARD OPERATING PROCEDURE		
Requires the signatures of the following persons:	Signature	Date: (dd/mmm/yy)
Author: Omara Denis<br>Laboratory Technologist		30-March-2022
Reviewed by: Dr Anne Kapaata<br>Viral Immunologist		30-March-2022
Authorized by: Dr Sheila N. Balinda<br>Molecular Virologist	SNBalinda	30-March-2022

REVISON HISTORY			
Version	Changes	Effective Date	Date Withdrawn
1.0	N/A	30-March-2022	

TABLE OF CONTENTS

1.0 PURPOSE ***3***

2.0 SCOPE ***3***

3.0 RESPONSIBILITIES ***3***

4.0 DEFINITIONS ***3***

5.0 REAGENTS AND MATERIALS ***5***

6.0 SAFETY PRECAUTIONS ***6***

7.0 INSTRUCTIONS AND PROCEDURES ***6***

7.1 Quality control measures **6**

7.2 Laboratory procedures **6**

7.2.1: Preparation of DNA from the Directly Cloned Recombinant Adenovirus Plasmid for Use in Transfection 6

7.2.2 Transfection of Packaging Cells with Linearized Adenovirus Vector DNA for Rescue 7

7.2.3 For Transfection Using Lipofectamine 7

7.2.4 For Calcium Phosphate Transfection 8

7.2.5 Expansion of Adenovirus Vector for Large-Scale Production 9

7.2.6 Troubleshooting 9

7.2.9 Discussion 10

7.2.10 Related Information 10

8.0 REFERENCES ***11***

9.0 Appendix ***12***

9.1 Appendix 1 Attachment of SOP Training document log **12**

9.2 Signature of responsible persons **13**

9.3 Competence evaluation for proper use of this SOP **14**

9.4 Competence certificate **16**

1.0 PURPOSE

This SOP describes transfection, infection, expansion, and large-scale production of modified Adenovirus using AD293 cell line (# 240085, Agilent). AD-293 cells, like HEK293 cells, produce the adenovirus E1 gene in trans, allowing the production of infectious virus particles when cells are transfected with E1-deleted adenovirus vectors such as the pAdEasy-1 vector. AD-293 cells demonstrate improved adherence to tissue culture dishes, making AD-293 cell monolayers less susceptible to disruption during cell passaging and plaque assays. This SOP describes every precaution and safety procedure that should be taken while conducting the assays.

2.0 SCOPE

This SOP should be followed by all personnel in the MRC/UVRI & LSHTM PGPI laboratories.
It provides instructions for conducting transfection, infection, expansion and large-scale production of modified Adenovirus using AD293 cell line in the CL3 laboratory. Specifically, it will be used within the NAV-COV19 vaccine project.

3.0 RESPONSIBILITIES

All personnel performing transfection, infection, expansion and large-scale production of modified Adenovirus using AD293 cell line at MRC/UVRI CL3 laboratory are responsible to comply with this SOP. The principal investigator is responsible for ensuring that all laboratory personnel is sufficiently trained to fully perform and implement this procedure. The author is responsible to review and revise this SOP.

4.0 DEFINITIONS

SOP	-	Standard operating procedures
CL3	-	Containment level three
MRC	-	Medical research council
UVRI	-	Uganda virus research institute
RPM	-	Revolution per minute
CPE	-	Cytopathic Effect

DNA - Deoxyribonucleic Acid

5.0 REAGENTS AND MATERIALS

Consumables and equipment

Sterile serological pipettes
Sterile pipette tipes with aerosol barrier
Disposable gloves
Gowns
Biosafety Level II tissue culture hood
Conical centrifuge bottles, sterile (150 and 500 mL)
Conical centrifuge tubes (15 and 50 mL)
Dry-ice/ethanol bath
Freezer (−80°C)
Gel electrophoresis apparatus
Humidified cell culture incubator (37°C, 5% CO2)
Microcentrifuge
Pasteur pipette attached to vacuum flask
Polystyrene round-bottomed tubes with snap caps (5 mL)
T-75 culture flasks
Tabletop centrifuge
Vortex machine
Water bath (37°C)

Reagents

Cesium Chloride (CsCl)
Light Cesium Chloride (L-CsCL)
Heavy Cesium Chloride (H-CsCL)
Dulbecco's minimal essential medium (DMEM)
Complete Growth Media (R10)
Agarose gel (1%)
CaCl2 (2 M)
Calcium Phosphate Transfection Kit (Promega ProFection Mammalian Transfection System)
Dulbecco's phosphate-buffered saline (D-PBS)
Fetal bovine serum (FBS)
HBS solution (2X)
HEK-293 A cells (American Type Culture Selection [ATCC]
Lipofectamine (Life Technologies)
PacI restriction enzyme and buffer
Penicillin–streptomycin (P/S) solution (100X)
Recombinant adenovirus plasmid (from Protocol: Construction of Recombinant Adenovirus Genomes by Direct Cloning [Zhou et al. 2019])
Tris (10 mM, pH 8.0)
Trypsin–EDTA (0.05%)
ZnSO4 (see Step 4.iv)

6.0 SAFETY PRECAUTIONS

Treat all specimens as potentially infectious. Universal precautions must be always adhered to at all times. Wear appropriate personal protective equipment (PPE) such as gloves, safety goggles, and lab coats. Procedures to ensure the health and safety of staff are outlined in SOPs HS-PPE-004, HS-SPR-001, HS-PEP-008, and HS-GSF-003.

According to the NIH Guidelines for Research Involving Recombinant DNA Molecules (April 2000), all types of wild-type and replication-competent adenoviruses are classified as risk group 2 of biohazard agents. The human disease associated with this group of biohazard agents is usually treatable and preventable and is rarely serious. All work with adenovirus vectors should be conducted at Biosafety Level 2 (BL2)

7.0 INSTRUCTIONS AND PROCEDURES

7.1 Quality control measures

Always include a negative and positive control

7.2 Laboratory procedures

7.2.1: Preparation of DNA from the Directly Cloned Recombinant Adenovirus Plasmid for Use in Transfection

It is critical to practice aseptic technique during the following preparation steps.

1. Digest the recombinant adenovirus plasmid with PacI endonuclease to release the intact adenovirus vector genome. The PacI sites flank both 5′ and 3′ ITRs located at the ends of the adenovirus vector genome. Set up the reaction as follows:

Recombinant adenovirus plasmid DNA	1 µg
PacI	0.2 µL
PacI buffer (10X)	1 µL
H2O	to 10 µL

Incubate the digest reaction for 60 minutes at 37°C.

2. Analyze 1µL of the reaction by electrophoresis on a 1% agarose gel to confirm successful release of the vector genome from the plasmid backbone. Mention the right sdize we are interested in.

7.2.2 Transfection of Packaging Cells with Linearized Adenovirus Vector DNA for Rescue

3. One day before transfection, seed early- or medium-passage HEK-293 cells (passage 31–60 for E1-deleted, E1/E3-deleted, and E1/E3-deleted+E4 orf6 adenovirus vectors) or 10-3 cells (for E1/E4- or E1/E3/E4-deleted adenovirus) or AD293 cells in 6-well tissue culture plates at 0.5 x106 cells per well.

4. On the next day, when cell confluency reaches ~50%–70%, proceed with one of the following transfection methods.

7.2.3 For Transfection Using Lipofectamine

No serum or antibiotics should be used.

i. For each transfection, label two 0.5-mL Eppendorf tubes as tubes A and B. Add 1 µg of PacI-digested recombinant adenovirus plasmid DNA to tube A and adjust the total volume to 60 µL with DMEM. Add 6.4 µL of Lipofectamine and 53.6 µL of DMEM to tube B. Gently mix the contents of each tube separately by swirling the tubes. Do not vortex. Combine the solutions of tube A and B using a wide-bore pipette. Gently mix them by swirling. Do not vortex. Let the mixture stand for 45 minutes at room temperature to form DNA–liposome complexes.

ii. At 5 minutes before the end of the incubation, gently rinse the cells once with DMEM prewarmed to 37°C and then add 3 mL of DMEM. Add the transfection mixture to the cells dropwise, rock the plate gently, and return the cells to the incubator. Three hours later, add 60 µL of FBS and incubate the cultures overnight.

iii. On the following day, replace the transfection medium with fresh Complete growth medium and continue to incubate the transfected cells. Add 200 µL of fresh Complete growth medium to the plate wells every 3 days and examine the cells for cytopathic effect (CPE).

CPE reflects the morphological changes of the cultured cells as infection proceeds. Such distinctive morphological changes result from the accumulation of newly produced virus progeny. CPE is different from necrosis; CPE begins with rounding of adhered cells followed by gradual detachment of the rounded cells from the plate. When the cells detach from the plate, they may form "grape-like" clusters that float in the growth medium.

iv. To rescue an adenovirus vector with a complete deletion of region E4, add 175 mM of ZnSO4 to the growth medium of 10-3 cells (in Step 4.iii).

The addition of $ZnSO_4$ is necessary to induce the metallothionein promoter that drives E4-orf6 expression in 10-3 cells (Gao et al. 1996).

7.2.4 For Calcium Phosphate Transfection

i. Replace the growth medium in the 6-well plate with 3 mL of fresh Complete growth medium prewarmed for at least 3 h to 37°C before transfection.

ii. Thaw all reagents in the calcium phosphate transfection kit at room temperature. Label as tubes A and B two polystyrene 5-mL tubes with snap caps. Aliquot 300 µL of 2X HBS solution into tube A and prepare 300 µL of DNA mix in tube B by adding 5 mg of PacI linearized clone of recombinant adenovirus plasmid DNA, 37.5 µL of 2 M $CaCl_2$, and the required volume of sterile H_2O. Mix the transfection solution by adding DNA mix in tube B into the 2X HBS in tube A dropwise while vigorously vortexing tube A. Incubate the transfection cocktail for 20 min at room temperature to form DNA–calcium phosphate precipitates. The solution should become translucent. After the incubation period, slowly add the transfection cocktail to the 6-well plate. Gently rock the plate to evenly distribute the DNA–calcium precipitates over the entire monolayer. Return the cells to the incubator.

iii. The next morning, wash cells once gently with DMEM prewarmed to 37°C and then add fresh Complete growth medium and continue the incubation. Add 1 mL of fresh growth medium to the plate every 3 days and examine the cells for CPE.

iv. To rescue an E4-deleted adenovirus vector, add 175 mM $ZnSO_4$ to the Complete growth medium of 10-3 cells.

Addition of $ZnSO_4$ is necessary to induce the metallothionein promoter that drives E4-orf6 expression in 10-3 cells.

5. Once 90% of the cell monolayer shows CPE, dislodge any remaining cells by gently tapping the plate on the work surface of the cell culture biosafety hood or pipetting the medium against the well growth surface. Transfer the cell suspension to a 15-mL conical centrifuge tube and store it at −80°C for virus expansion.

If the transfected cells do not show any sign of CPE 2 weeks after the transfection, see Troubleshooting.

7.2.5 Expansion of Adenovirus Vector for Large-Scale Production

6. 2.3.1. Seed 10 X 106 AD-293 cells into a T75 cell culture flask 1 day before viral infection.

i. To prepare a crude cell lysate, thaw the cell suspension from Step 5 in a 37°C water bath. Repeat the freeze–thaw cycle twice more in a dry-ice/ethanol bath and a 37°C water bath. Shake the tube several times after each thaw to ensure that cells do not settle.

ii. Centrifuge the cells at 3200 rpm in a tabletop centrifuge for 10 minutes at 4°C.

iii. Remove the supernatant and add it directly to two 70%–80% confluent T75 cell culture flask of AD293 cells. Return the cells to the incubator and monitor CPE on a daily basis.

Usually, CPE will become noticeable 24 h after infection and will be fully evident within 2–3 days.

7. Harvest the cells when 90% of the cells show CPE, as before, and centrifuge at 3200 rpm for 10 min at 4°C. Discard the supernatant and resuspend the cell pellet in 2% complete DMEM. After three cycles of freezing/thawing, centrifuge the crude cell lysate at 3200 rpm for 10 min at 4°C and collect the supernatant for infection of eight plates of HEK-293 cells, as in Step 6.

8. At ~40–45 h after infection, or when 90% of the infected cells show CPE, harvest the cells as before and centrifuge at 3200 rpm for 10 min at 4°C. Resuspend the cell pellet in 8 mL of 2% complete DMEM and store at −80°C.

i. For expansion of the E4-deleted adenovirus vectors, use 10-3 cells and supplement the Complete growth medium with 175 mM ZnSO4 when sending the cells to induce expression of the E4 orf6 necessary for replication of this vector.

If the crude viral lysate harvested from the transfection/rescue step fails to expand after 7 d, see Troubleshooting (7.2.8).

7.2.6 Troubleshooting

Problem (Step 5): Transfected HEK-293 cells do not show any sign of CPE 2 weeks after transfection.

Solutions: Repeat the transfection using 7.5 mg of linearized recombinant adenovirus plasmid DNA.

Use a different transfection method.

• Extend the observation time up to 4 weeks after transfection.

• If the transgene is known to be cytotoxic or cytostatic, introduce a gene regulation mechanism into the transgene expression cassette.

Problem (Step 8): The crude viral lysate fails to expand in HEK-293 (or 10-3) cells.

Solution: There are several possible causes for this problem, including cytotoxicity associated with transgene expression, which leads to cell death instead of viral CPE; an oversized transgene cassette; the nature and numbers of early gene deletions in the vector backbone (i.e., E1 deletion only, E1/E3 deletions, or E1/E3/E4 deletions); levels of complementing gene expression in the cell line (e.g., 10-3 cells); and non-synchronized viral infection caused by a low MOI. In the last case, when the cells are infected at low MOIs, in order to reach full CPE, serial passage of the virus stock may be required. For vectors that have a slow and delayed infection process, it can be difficult to distinguish cell death from viral CPE. The following strategies should be considered for overcoming this problem.

• Construct an inducible transgene expression cassette to regulate expression of the toxic transgene in 293 cells.

• Slow down the expansion process. Start the expansion from the crude lysate of the original transfection/rescue to one 150-mm plate of 293 cells to 3 plates, 12 plates, and finally 40 plates of 293 cells.

• In the expansion process, if the cells do not reach full CPE in 72 h, prepare, and clarify the crude
lysate from the entire infection (not from the cell pellet) for the next stage of infection.

7.2.9 Discussion

As shown in Figure 1 of Protocol: Construction of Recombinant Adenovirus Genomes by Direct Cloning (Zhou et al. 2019), for nontoxic and nonoversized transgenes, rescue of virus from a recombinant adenovirus plasmid and expansion of the rescued infectious viral vector for large-scale production should be accomplished in _3 wk. Conversely, for recombinant adenovirus plasmids oversized or toxic transgenes, viral vector rescue and expansion steps may take up to 12 wk.

The AD293 cell line is a subclone of the 293 cell line with a relatively flat morphology. It facilitates the initial production, amplification, and titration of replication-deficient adenoviruses. The cell line contains a stably integrated copy of the E1 gene that provides the E1 proteins (E1a and E1b) required for the production of recombinant adenovirus. The flat morphology of the cells makes the titration procedure simpler.

7.2.10 Related Information

According to the NIH Guidelines for Research Involving Recombinant DNA Molecules (April 2000),

all types of wild-type and replication-competent human adenoviruses are classified as risk group 2 of

biohazard agents. The human disease associated with this group of biohazard agents is usually treatable and preventable and is rarely serious. All work with adenovirus vectors should be conducted at Biosafety Level 2 (BL2) with the approval by the Institutional Biosafety Committee of the home institution.

RECIPES

Complete Growth Medium

Reagent	Quantity (for 1 L)	Final concentration
DMEM	890 mL	
Fetal bovine serum (FBS)	100 mL	10%
Penicillin/streptomycin (P/S) solution (100×)	10 mL	1×

Store at 4°C.

HBS Solution (2×)

Reagent	Quantity (1 L)	Final concentration
NaCl	16.4 g	280 mM
HEPES ($C_8H_{18}N_2O_4S$)	11.9 g	50 mM
$Na_2HPO_4 \cdot 7H_2O$	0.38 g	1.42 mM
H_2O	to 1 L	

Adjust pH to 7.05 with 10 M NaOH. Sterilize by passing through a 0.22-µm filter and store at room temperature.

8.0 REFERENCES

1. Gao GP, Yang Y, Wilson JM. 1996. Biology of adenovirus vectors with E1 and E4 deletions for liver-directed gene therapy. J Virol 70: 8934–8943.
2. NIH Guidelines for Research Involving Recombinant DNA Molecules. April 2000.
3. Su Q, Sena-Esteves M, Gao G. 2019. Purification of the recombinant adenovirus by cesium chloride gradient centrifugation. Cold Spring Harb Protoc doi: 10.1101/pdb.prot095547.
4. Zhou X, Sena-Esteves M, Gao G. 2019. Construction of recombinant adenovirus genomes by direct cloning. Cold Spring Harb Protoc doi: 10.1101/pdb.prot095521.

9.0 Appendix

9.1 Appendix 1 Attachment of SOP Training document log

Name	Signature	Date	Trainer

9.2 Signature of responsible persons

"I have read and understand this SOP. I agree to fully adhere to its requirements."

Date	Name	Signature

9.3 Competence evaluation for proper use of this SOP

Trainee: ____________________________ Assessor: ____________________

Did the trainee do the following correctly? COMMENTS

1	Did you read the SOP which applies to Transfection, infection, expansion, and large-scale production of modified Adenovirus using AD293 cell line.	☐ YES ☐ No	
2	Ensured all the necessary equipment and reagents are available and used for the procedure	☐ YES ☐ No	
3	Switched on water bath at 37°c	☐ YES ☐ No	
4	Thawed the cells rapidly	☐ YES ☐ No	
5	Added recommended volume of prewarmed growth medium and cultured in a CO_2 incubator.	☐ YES ☐ No	
6	Resuspend the cell with cDMEM	☐ YES ☐ No	
7	Counted the cells with trypan blue	☐ YES ☐ No	
8	Have the cell attained recommended confluency after 24 hours incubation?	☐ YES ☐ No	
9	Have the cells been trypsinised well?	☐ YES ☐ No	
10	Was the Transfection, infection, expansion, and large-scale production of modified Adenovirus using AD293 cell line successful?	☐ YES ☐ No	

Observer Notes:	
Is the trainee competent to use the machine?	☐ YES ☐ No
Remedial action taken:	Date complete: ____________

Signatures:

Assessor/Date:____________________ Manager/Reviewer/Date:_____________________

NAV-COV-19 Study Programme Head/Date:__________________________

9.4 Competence certificate

Certificate of competence

This is to certify that

……………………………………………….

has read the “transfection, infection, expansion and large-scale production of modified Adenovirus using AD293 cell line” SOP training and has been trained on how to use the machine. He/she is now competent to the assays without any supervision.

1. *Trainer*

Name& tittle………………………………………………………………………………

Signature…………………………………………..Date…………………………………

2. *Approved by*

Name& title………………………………………………………………………………

Signature…………………………………………..Date…………………………………
