# Supplementary material for: Practical guidelines for producing non-replicating canine adenovirus vectors
Source: PLoS One. 2026 May 20;21(5):e0341642. doi: 10.1371/journal.pone.0341642 (PMC13189411; doi:10.1371/journal.pone.0341642)
Supplement: S2 File — (PDF) [file pone.0341642.s006.pdf]

MRC/UVRI and LSHTM Uganda Research Unit

DO NOT COPY

Written by:	Name: Omara Denis<br>Function/role: Laboratory technologist

APPROVAL OF STANDARD OPERATING PROCEDURE		
Requires the signatures of the following persons:	Signature	Date: (dd/mmm/yy)
Author: Omara Denis<br>Laboratory Technologist		30-March-2022
Reviewed by: Dr Anne Kapaata<br>Viral Immunologist		30-March-2022
Authorized by: Dr Sheila N. Balinda<br>Molecular Virologist		30-March-2022

REVISON HISTORY			
Version	Changes	Effective Date	Date Withdrawn
1.0	N/A	30-March-2022	

TABLE OF CONTENTS

1.0 PURPOSE *3*

2.0 SCOPE 3

3.0 RESPONSIBILITIES 3

4.0 DEFINITIONS *3*

5.0 REAGENTS AND MATERIALS *4*

6.0 SAFETY PRECAUTIONS *4*

7.0 INSTRUCTIONS AND PROCEDURES *4*

7.1 Quality control measures 4

7.2 Laboratory procedures 5

7..2.1 Sample preparation 5

7.2.2 Vivapure Q Maxi M preparation 5

7.2.3 Adenovirus purification 6

7.2.4 Optional: Buffer exchange and further concentration 6

7.2.5: Typical performance 7

7.2.6: Usage tips 7

8.0 REFERENCES *7*

9.0 Appendix *8*

9.1 Appendix 1 Attachment of SOP Training document log 8

9.2 Signature of responsible persons 8

9.3 Competence evaluation for proper use of this SOP 10

Appendix 4: Competence certificate 12

1.0 PURPOSE

This SOP describes the purification of Adenovirus with Vivapure Q Maxi M spin columns containing an ion exchange membrane adsorber that binds adenoviral particles. Once bound, virus particles can be further purified by washing away non-specifically bound proteins before elution within one hour.The Vivapure Q Maxi M is designed for parallel purification and concentration of adenovirus from 20 ml culture medium. Ready-to-use filter devices, Vivapure Q Maxi M spin columns, centrifugal Vivaspin concentrators and buffers make the following purification procedure as easy as filtration.

2.0 SCOPE

This SOP should be followed by all staff members conducting Vivapure Adenopack 20 RT Virus Purification in the CL3 laboratory. Specifically, it will be used within the NAV-COV19 vaccine project.

3.0 RESPONSIBILITIES

All personnel performing Vivapure Adenopack 20 RT virus purification technique at MRC/UVRI CL3 laboratory are responsible to comply with this SOP. The principal investigator is responsible for ensuring that all laboratory personnel is sufficiently trained to fully perform and implement this procedure. The author is responsible to review and revise this SOP.

4.0 DEFINITIONS

SOP	-	Standard operating procedures
CL3	-	Containment level three
MRC	-	Medical research council
UVRI	-	Uganda virus research institute
RPM	-	Revolution per minute
CPE	-	Cytopathic Effect
DNA	-	Deoxyribonucleic Acid
Ad	-	Adenovirus

5.0 REAGENTS AND MATERIALS

Consumables and equipment

Biosafety Level II tissue culture hood

Pasteur pipette attached to vacuum flask

Centrifuge with swing-out rotors accepting 50 ml falcon tubes

Water bath at 25°C

Sterile plastic container for sample handling

Reagents

Vivapure Adenopack 20 RT Kit

Adenovirus to be Quantified

Ethanol dry ice bath or -80°C freezer

1500 U Benzonase® Nuclease

Optional – Storage Buffer: 20 mM Tris/HCl, 25 mM NaCl, 2.5% Glycerol (w/v), pH 8.0 at 22°C

6.0 SAFETY PRECAUTIONS

Treat all specimens as potentially infectious. Universal precautions must be always adhered to at all times. Wear appropriate personal protective equipment (PPE) such as gloves, safety goggles, and lab coats. Procedures to ensure the health and safety of staff are outlined in SOPs HS-PPE-004, HS-SPR-001, HS-PEP-008, and HS-GSF-003.

According to the NIH Guidelines for Research Involving Recombinant DNA Molecules (April 2000), all types of wild-type and replication-competent adenoviruses are classified as risk group 2 of biohazard agents. The human disease associated with this group of biohazard agents is usually treatable and preventable and is rarely serious. All work with adenovirus vectors should be conducted at Biosafety Level 2 (BL2)

7.0 INSTRUCTIONS AND PROCEDURES

7.1 Quality control measures

Always include a negative and positive control

7.2 Laboratory procedures

7..2.1 Sample preparation

Note: *Each Vivapure Q Maxi M spin column may be used to purify virus from up to 20 ml culture volume. This kit contains sufficient consumables for six such small preparations. Please adjust reagent volumes accordingly for smaller samples.*

1. Amplify adenovirus in low passage HEK 293 cells in up to 20 ml total culture volume (E.g., one 15 cm plate with 20 ml culture) that has been infected with an adenovirus stock at an MOI of 10–20. Cultures should be grown in DMEM +10% FBS pH 7.0–7.4 at 37°C with 5% CO2.
2. Once most of the cells show cytopathic effects (2–5 days), pool cells and medium. It may be necessary to detach adhering cells using a pipette or cell scraper.
3. Centrifuge at 3,500 xg for 15 minutes to pellet cells.
4. Decant supernatant to a sterile container and set aside.
5. Re-suspend cell pellet in 2 ml supernatant.
6. Freeze – thaw the cell suspension completely 3 times to disrupt cells alternately using a 25°C water bath and ethanol/dry-ice bath or –80°C freezer.
7. Centrifuge at 3,500 + g for 15 minutes to pellet cell debris.
8. Decant viral supernatant, re-combine it with the original supernatant and mix gently.
9. Add Benzonase® Nuclease, to a final concentration of 12.5 U/ml.
10. Mix sample and incubate for 30 minutes at 37°C in order to digest cellular nucleic acids.
11. Load digested supernatant on a Vivapure Maxi and spin the device 5 minutes at 500 xg or until the whole volume has passed the membrane.
12. Collect the flow-through. Estimate the volume and slowly add 1/9 volume of 10-fold loading buffer under agitation to avoid osmotic shock in the virus particles. E.g., 2 ml to 18 ml flow-through. Accurate volume measurements of the supernatant and 10X Loading Buffer are critical to achieving the right conditions for binding virus particles.

7.2.2 Vivapure Q Maxi M preparation

13. Dilute 10 x Washing Buffer to working concentration, e.g., for one preparation: 5 ml buffer with 45 ml deionised water and mix well.

14. Equilibrate the Vivapure Q Maxi M spin column with 5 ml diluted Washing Buffer and spin for 5 minutes at 500 xg. We recommend the use of a swing-out rotor to ensure the uniform flow of your sample solution.

7.2.3 Adenovirus purification

15. Load the sample (no more than 20 ml) into the Vivapure Q Maxi M insert and spin the device for 5 min at 500 xg or until the whole volume has passed the membrane. Collect flow-through and repeat the step with residual sample if necessary.
16. Wash the spin column with 18 ml Washing Buffer byspinning for 5 minutes at 500 xg. Remove flow-through and repeat the wash step once.
17. Using a fresh collection tube, elute adenovirus off the Vivapure Q Maxi M membrane using 1 ml Elution Buffer. Pipette buffer onto the membrane, centrifuge for 30 seconds at 500 xg and incubate 10 minutes. Then spin the device for 5 min at 500 x g and collect the adenovirus-containing eluate. Applying a second elution step could improve yield but will dilute virus titre.

7.2.4 Optional: Buffer exchange and further concentration

Note: *It is necessary that virus is exchanged into physiological buffer before use in tissue culture or cell-based assays, or into generic Storage Buffer for long-term storage at -80°C. Storage Buffers containing glycerine may take considerably longer to concentrate than the original viral eluate solution; prolong centrifuge times and if necessary use cooling at +4°C.*

18. Transfer eluate to a Vivaspin 20 centrifugal concentrator and add storage/physiological buffer to the concentrate to bring the volume up to 10 ml. Counterbalance the rotor with a second concentrator filled with an equivalent volume of PBS or water. In fixed angle rotors the printed graduations should face away from the centre of the rotor.
19. Centrifuge for 30 minutes at up to 800 + g in a swing-out rotor, or 25° fixed-angle rotor, with cavities accepting 50 ml conical bottom tubes.
20. Check the volume of viral concentrate remaining in the upper chamber and if necessary, centrifuge again and repeat buffer exchange a second time.

Caution: *Do not reduce the volume to less than 200 µl in order to avoid aggregation and loss of infectivity.*

21. Recover the concentrated virus by pipette. Resuspend concentrated virus by gently pipetting up and down a few times before recovery.

22. Determine viral titre. Aliquot accordingly and store virus at –80°C. Storage buffers for Adenovirus are to be found on page 3 and in the following publication: (Hoganson, D. K. et al., 2003).

7.2.5: Typical performance

For a normal yielding vector, 1+15 cm culture plates purified using this method should yield a range of up to $1x10^{12}$ viral particles (see table 1).

7.2.6: Usage tips

– It is recommended that virus is exchanged into normal physiological buffer before use in tissue culture or cellbased assays.

– Aliquot and store virus at -80°C. Once thawed, keep at +4°C and do not re-freeze.

– Virus should remain viable for up to 2 years at -80°C when purified by this procedure.

Table1: Purification results from preparations with certain Adenovirus GFP-constructs – depending on individual conditions values may be different.

Purification method	Process time	Eluate	Recovery**	Viral parti- cles
20 ml culture	1 hour	1 ml	65–70%	$1 \times 10^{11-12}$
500 ml CsCl	12–48 hours	1–2* ml	60–70%	$1 \times 10^{11-12}$

* after dialysis
** before buffer exchange

8.0 REFERENCES

1. Hoganson, D. K. et al., Development of a Stable Adenoviral Vector Formulation (2003), Bioprocessing Journal, pp. 43-48.

2. Capone, S., Raggioli, A., Gentile, M., Battella, S., Lahm, A., Sommella, A., ... & Vitelli, A. (2021). Immunogenicity of a new gorilla adenovirus vaccine candidate for COVID-19. *Molecular Therapy*, *29*(8), 2412-2423.
3. Lee, Soo Young, et al. "CD44-shRNA recombinant adenovirus inhibits cell proliferation, invasion, and migration, and promotes apoptosis in HCT116 colon cancer cells." *International journal of oncology* 50.1 (2017): 329-336.

9.0 Appendix

9.1 Appendix 1 Attachment of SOP Training document log

Name	Signature	Date	Trainer

9.2 Signature of responsible persons

"I have read and understand this SOP. I agree to fully adhere to its requirements."

Date	Name	Signature

9.3 Competence evaluation for proper use of this SOP

Trainee: ____________________________ Assessor: ____________________

Did the trainee do the following correctly? COMMENTS

1	Did you read the SOP which applies to plaque assay to quantify adenovirus.	☐ YES ☐ No	
2	Ensured all the necessary equipment and reagents are available and used for the procedure	☐ YES ☐ No	
3	Switched on water bath at 37°c	☐ YES ☐ No	
4	Plated the right cell concentration	☐ YES ☐ No	
5	Added recommended volume of prewarmed growth medium and cultured in a CO_2 incubator.	☐ YES ☐ No	
6	Resuspend the cell with cDMEM	☐ YES ☐ No	
7	Counted the cells with trypan blue	☐ YES ☐ No	
8	Have the cell attained recommended confluency after 24 hours incubation?	☐ YES ☐ No	
9	Have you stained the cells with the right stain?	☐ YES ☐ No	
10	Are you using the right formular to calculate the Plaque Forming Units?	☐ YES ☐ No	

Observer Notes:	
Is the trainee competent to use the machine?	☐ YES ☐ No
Remedial action taken:	Date complete: ____________

Signatures:
Assessor/Date:____________________ Manager/Reviewwer/Date:_____________________

NAV-COV-19 Study Programme Head/Date:__________________________

Appendix 4: Competence certificate

Certificate of competence

This is to certify that

……………………………………………….

has read the "Vivapure Adenopack 20 RT Virus Purification" SOP training and has been trained on how to use the machine. He/she is now competent to the assays without any supervision.

1. *Trainer*

Name& tittle……………………………………………………………………………

Signature…………………………………………..Date…………………………………

2. *Approved by*

Name& title……………………………………………………………………………

Signature…………………………………………..Date…………………………………
