# Supplementary material for: Practical guidelines for producing non-replicating canine adenovirus vectors
Source: PLoS One. 2026 May 20;21(5):e0341642. doi: 10.1371/journal.pone.0341642 (PMC13189411; doi:10.1371/journal.pone.0341642)
Supplement: S3 File — (PDF) [file pone.0341642.s007.pdf]

MRC/UVRI and LSHTM Uganda Research Unit

Medical Research Council

Uganda Virus Research Institute

LONDON SCHOOL of HYGIENE &TROPICAL MEDICINE

SOP virus titration using Improved Kärber Method	Version 1.0	Effective Date 30-March-22

DO NOT COPY

Written by:	Name: Omara Denis<br>Function/role: Laboratory technologist

APPROVAL OF STANDARD OPERATING PROCEDURE		
Requires the signatures of the following persons:	Signature	Date: (dd/mmm/yy)
Author: Omara Denis<br>Laboratory Technologist	[signature]	30-March-2022
Reviewed by: Dr Anne Kapaata<br>Viral Immunologist	[signature]	30-March-2022
Authorized by: Dr Sheila N. Balinda<br>Molecular Virologist	[signature]	30-March-2022

REVISON HISTORY			
Version	Changes	Effective Date	Date Withdrawn
1.0	N/A	30-March-2022	

TABLE OF CONTENTS

1.0 PURPOSE *3*

2.0 SCOPE 3

3.0 RESPONSIBILITIES 3

4.0 DEFINITIONS *3*

5.0 REAGENTS AND MATERIALS *4*

6.0 SAFETY PRECAUTIONS *4*

7.0 INSTRUCTIONS AND PROCEDURES *5*

7.1 Cell preparation, virus serial dilution and CPE observation 5

7.2 Calculation of 50% Tissue Culture Infectious Dose (TCID50) of the titered virus stocks 5

7.3 A typical example of a titer plate. 6

8.0 REFERENCES *7*

9.0 Appendix *8*

9.1 Appendix 1 Attachment of SOP Training document log 8

9.2 Signature of responsible persons 9

9.3 Competence evaluation for proper use of this SOP 10

9.4: Competence certificate 12

1.0 PURPOSE

This SOP describes the determination of virus stock titer by determining the TCID50. In this protocol, the purified virus stock was titered using the Improved Kärber method. The Improved Kärber method involves preparing a monolayer of AD-293 cells in 96-well plates, followed by a series of dilutions of the virus stock. The endpoint, where 50% of wells show CPE, helps calculate the tissue culture infectious dose (TCID50).

2.0 SCOPE

This SOP should be followed by all staff members conducting virus stock titration in the CL3 laboratory. Specifically, it will be used within the NAV-COV19 vaccine project.

3.0 RESPONSIBILITIES

All personnel performing virus stock titration technique at MRC/UVRI CL3 laboratory are responsible to comply with this SOP. The principal investigator is responsible for ensuring that all laboratory personnel is sufficiently trained to fully perform and implement this procedure. The author is responsible to review and revise this SOP.

4.0 DEFINITIONS

SOP	-	Standard operating procedures
CL3	-	Containment level three
MRC	-	Medical research council
UVRI	-	Uganda virus research institute
RPM	-	Revolution per minute
CPE	-	Cytopathic Effect
DNA	-	Deoxyribonucleic Acid
Ad	-	Adenovirus

5.0 REAGENTS AND MATERIALS

Consumables and equipment

Biosafety Level II tissue culture hood

Pasteur pipette attached to vacuum flask

Centrifuge with swing-out rotors accepting 50 ml falcon tubes

Water bath at 25°C

Sterile plastic container for sample handling

Inverted microscope

96-flat bottom well tissue culture plate

Reagents

AD-293 cell line

DMEM-2% FBS

Pen-strep antibiotics

Hepes Buffer

L-glutamine solution

6.0 SAFETY PRECAUTIONS

Treat all specimens as potentially infectious. Universal precautions must be always adhered to at all times. Wear appropriate personal protective equipment (PPE) such as gloves, safety goggles, and lab coats. Procedures to ensure the health and safety of staff are outlined in SOPs HS-PPE-004, HS-SPR-001, HS-PEP-008, and HS-GSF-003.

According to the NIH Guidelines for Research Involving Recombinant DNA Molecules (April 2000), all types of wild-type and replication-competent adenoviruses are classified as risk group 2 of biohazard agents. The human disease associated with this group of biohazard agents is usually treatable and preventable and is rarely serious. All work with adenovirus vectors should be conducted at Biosafety Level 2 (BL2)

7.0 INSTRUCTIONS AND PROCEDURES

7.1 Cell preparation, virus serial dilution and CPE observation

1. A day before, trypsinise and count AD-293 cells. Then prepare 20 ml of 1000 cells/ml in 10% cDMEM culture medium.
2. Dispense 100µl (~100 cells) per well in two 96-well plates per sample and put them in an incubator.
3. On the following day, prepare virus dilutions by adding 108µl of 10% cDMEM to all the wells of 96 U-bottom dilution plates from column 1 to column 10 for ten replicas. Columns 11 and 12 are control columns, where 100uL of culture medium should be added.
4. Retrieve purified virus stocks from -80 ^{0}C freezer, thaw and add 12uL of the virus stock to the first row to make 10^{-1} (10-fold) dilution.
5. Using a multichannel pipette, serially dilute the virus stock by transferring 12uL from row 1 (10^{-1}) to row 2 (10^{-2}) and continue up to row 16 (10^{-16}) on the second plate.
6. Retrieve the culture plates that have already attained about 70% confluency from the incubator and remove the medium from the wells.
7. Transfer 100µL of the diluted virus from the dilution plate to the culture plate in their respective wells and incubate at 37°C with 5% CO_2.
8. Score wells for cytopathic effects (CPE) by counting the wells that have shown CPE (Figure 2) daily until there is no more CPE formation for two consecutive days. For the experiment to be valid, all the control wells must show no CPE.

7.2 Calculation of 50% Tissue Culture Infectious Dose (TCID50) of the titered virus stocks

1. Calculate the titres by determining the ratio of positive wells per row, as shown in **Error! Reference source not found.**, and use the Improved Kärber formula to determine the TICD50.

$$\text{Improved Kärber Formular} = \log \text{TCID}_{50} = \log d_L - \log d_F \left(\sum p_i - 0.5\right)$$

Where:
d_L = lowest dilution at which all wells are positive)
d_F = dilution factor
$\sum p_i$ = sum of the proportion of positive wells for all dilutions showing CPE

7.3 A typical example of a titer plate.

Dilutions	Sample										Control	
	1	2	3	4	5	6	7	8	9	10	11	12
10^{-1}	CPE	CPE	CPE	CPE	CPE	CPE	CPE	CPE	CPE	CPE		
10^{-2}	CPE	CPE	CPE	CPE	CPE	CPE	CPE	CPE	CPE	CPE		
10^{-3}	CPE	CPE			CPE	CPE		CPE		CPE		
10^{-4}		CPE				CPE						
10^{-5}												
10^{-6}												
10^{-7}												
10^{-8}												
10^{-9}												
10^{-10}												
10^{-11}												
10^{-12}												
10^{-13}												
10^{-14}												
10^{-15}												
10^{-16}												

In this case:
d_L =
dF = 10-fold
$\sum p_i$ = sum of the proportion of positive wells for all dilutions showing CPE of which in this case is 2.8
$logTCID_{50} = log10^{-2} – log10\ (2.8-0.5)$
$logTCID_{50} = -2 – 1\ (2.3)$
$logTCID_{50} = -4.3$
$TCID_{50}$ /100µl = $log10^{-4.3}$
$TCID_{50}$ /mL = 2.0×10^5

8.0 REFERENCES

1. Lei, C., Yang, J., Hu, J., & Sun, X. (2021). On the calculation of TCID50 for quantitation of virus infectivity. *Virologica Sinica, 36*(1), 141-144.

9.0 Appendix

9.1 Appendix 1 Attachment of SOP Training document log

Name	Signature	Date	Trainer

9.2 Signature of responsible persons

"I have read and understand this SOP. I agree to fully adhere to its requirements."

Date	Name	Signature

9.3 Competence evaluation for proper use of this SOP

Trainee: ______________________________ Assessor: _____________________

Did the trainee do the following correctly? COMMENTS

1	Did you read the SOP which applies to plaque assay to quantify adenovirus.	☐ YES ☐ No	
2	Ensured all the necessary equipment and reagents are available and used for the procedure	☐ YES ☐ No	
3	Switched on water bath at 37°c	☐ YES ☐ No	
4	Plated the right cell concentration	☐ YES ☐ No	
5	Added recommended volume of prewarmed growth medium and cultured in a CO_2 incubator.	☐ YES ☐ No	
6	Resuspend the cell with cDMEM	☐ YES ☐ No	
7	Counted the cells with trypan blue	☐ YES ☐ No	
8	Have the cell attained recommended confluency after 24 hours incubation?	☐ YES ☐ No	
9	Have you stained the cells with the right stain?	☐ YES ☐ No	
10	Are you using the right formular to calculate the Plaque Forming Units?	☐ YES ☐ No	

Observer Notes:
Is the trainee competent to use the machine? ☐ YES ☐ No
Remedial action taken: Date complete: _____________

Signatures:

Please ensure that you have an up-to-date version.

Assessor/Date:____________________ Manager/Reviewwer/Date:_____________________

NAV-COV-19 Study Programme Head/Date:__________________________

9.4: Competence certificate

Certificate of competence

This is to certify that

………………………………………………

has read the "Vivapure Adenopack 20 RT Virus Purification" SOP training and has been trained on how to use the machine. He/she is now competent to the assays without any supervision.

1. *Trainer*

Name& tittle………………………………………………………………………………

Signature…………………………………………..Date…………………………………

2. *Approved by*

Name& title………………………………………………………………………………

Signature…………………………………………..Date…………………………………
